# Age-related transcriptional modules and TF-miRNA-mRNA interactions in neonatal and infant human thymus

**DOI:** 10.1371/journal.pone.0227547

**Published:** 2020-04-15

**Authors:** Fernanda Bernardi Bertonha, Silvia Yumi Bando, Leandro Rodrigues Ferreira, Paulo Chaccur, Christiana Vinhas, Maria Claudia Nogueira Zerbini, Magda Maria Carneiro-Sampaio, Carlos Alberto Moreira-Filho

**Affiliations:** 1 Department of Pediatrics, Faculdade de Medicina da Universidade de São Paulo, São Paulo, SP, Brazil; 2 Instituto Dante Pazzanese de Cardiologia, São Paulo, SP, Brazil; 3 Department of Pathology, Faculdade de Medicina da Universidade de São Paulo, São Paulo, SP, Brazil; University of Pittsburgh, UNITED STATES

## Abstract

The human thymus suffers a transient neonatal involution, recovers and then starts a process of decline between the 1^st^ and 2^nd^ years of life. Age-related morphological changes in thymus were extensively investigated, but the genomic mechanisms underlying this process remain largely unknown. Through Weighted Gene Co-expression Network Analysis (WGCNA) and TF-miRNA-mRNA integrative analysis we studied the transcriptome of neonate and infant thymic tissues grouped by age: 0–30 days (A); 31days-6 months (B); 7–12 months (C); 13–18 months (D); 19-31months (E). Age-related transcriptional modules, hubs and high gene significance (HGS) genes were identified, as well as TF-miRNA-hub/HGS co-expression correlations. Three transcriptional modules were correlated with A and/or E groups. Hubs were mostly related to cellular/metabolic processes; few were differentially expressed (DE) or related to T-cell development. Inversely, HGS genes in groups A and E were mostly DE. In A (neonate) one third of the hyper-expressed HGS genes were related to T-cell development, against one-twentieth in E, what may correlate with the early neonatal depletion and recovery of thymic T-cell populations. This genomic mechanism is tightly regulated by TF-miRNA-hub/HGS interactions that differentially govern cellular and molecular processes involved in the functioning of the neonate thymus and in the beginning of thymic decline.

## Introduction

The human thymus grows only during the first year of life and its steady involution begins thereafter[1]. Moreover, the human thymus presents some functional peculiarities in the neonatal period and along the first six months of age, i.e. during minipuberty, when a transient surge in gonadal hormones takes place[2]. In the neonatal thymus occurs a transient involution marked by severe depletion of double-positive (DP) thymocytes, which is later compensated by increased levels of primitive T-cell precursors. Concomitantly, there is a reinforcement of the subcapsular epithelial cell layer and an increase of the intralobular extracellular matrix network, leading to augmented thymic permeability and to the recirculation of primitive precursors and mature T-cells in the neonatal thymus[3]. During minipuberty sex differences in thymic tissue were detected by gene co-expression network analysis for differentially expressed genes and by miRNA-target analysis, but such dimorphism vanishes after the 6^th^ month of age[4]. After the first year of life the total amount of lymphatic thymic tissue declines 5% per year until the 10^th^ year and at progressively slower rates afterwards[1]. The histomorphological features of thymic post-natal growth and of infant and adult thymic aging (lymphatic tissue declines, lipomatous atrophy) were quite well studied[1, 5], but the genomic mechanisms underlying these processes remain poorly understood.

The early programming of the thymus—sexual dimorphism, dynamics of thymocyte populations, thymic microenvironment changes—determines immune activity throughout life[4–7]. Therefore, we decided to perform a comparative transcriptome analysis of whole thymic tissue (cardiac surgery explants) from human neonates and from infants grouped according to sequential age intervals (6 months) up to the first 2 ½ years of life. Whole thymic tissue transcriptome datasets were interpreted through modular repertoire identification, an approach that has been used for investigating immune responses *in vivo* following the administration of vaccines[8–11]. Here we employed a weighted gene co-expression network analysis (WGCNA)[12] for describing correlation patterns among genes across microarray datasets that allows: i) the identification of transcriptional modules[10] and their association to particular age groups; ii) the identification of highly connected genes (hubs) and of significant genes (HGS genes) for the trait of interest (age). This analysis was complemented by an integrative mRNA-miRNA-Transcription Factor (TF) co-expression analysis encompassing: mRNAs from hubs and HGS genes, the abundantly expressed miRNAs, and the TFs that covaried with hubs and/or HGS genes.

## Results

**Fig 1:**
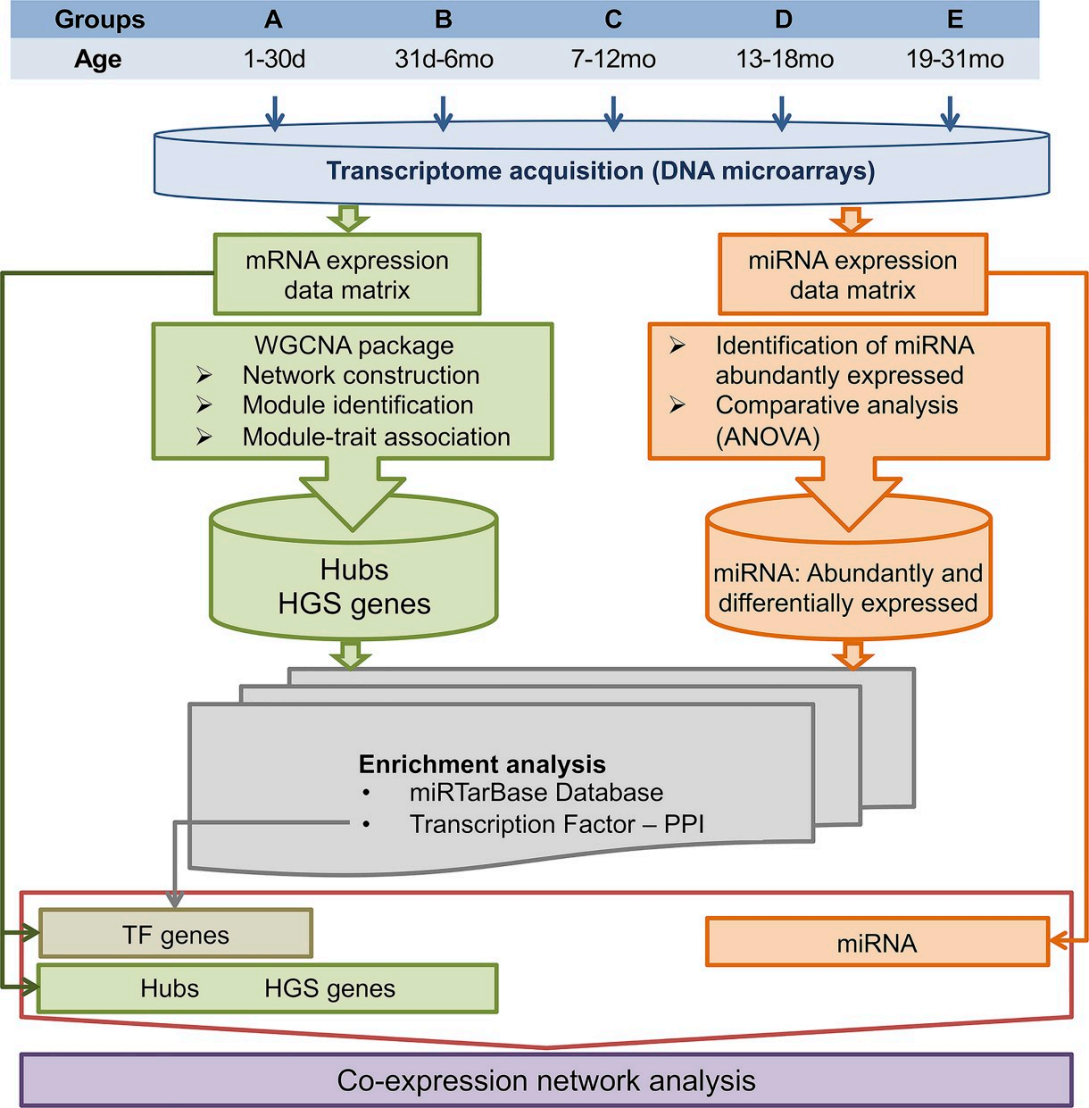
Workflow of the experimental approach and overview of the bioinformatic analyses.

Thymic tissue samples were obtained from 57 karyotypically normal patients who underwent cardiac surgery. For genomic analyses samples were classified according to patients’ age in five sequential age groups: A, neonates up to 30 days; B, infants aged 31 days to six months; C, infants aged 7–12 months; D, infants aged 13–18 months; and E, patients aged 19–31 months (S1 Table). The genomic analyses were centered in the identification of transcriptional modules by WGCNA and in mRNA-miRNA-Transcription Factor (TF) co-expression network analysis. The experimental workflow is depicted in Fig 1.

### TMA-IHC analysis

Tissue microarray and immunohistochemistry techniques (TMA-IHC) were used for quantification of specific cortical and medullar thymic cell subpopulations–CD4+ and CD8+ thymocytes, CD20+ B-cells and AE1/AE3 epithelial cells–showing the expectable moderate increase of thymocyte and B-cell populations and the overall maintenance of epithelial cell populations along the first two years of age (S1 Fig), as previously described[1, 3].

### Weighted Gene Co-expression Network Analysis (WGCNA)

**Fig 2:**
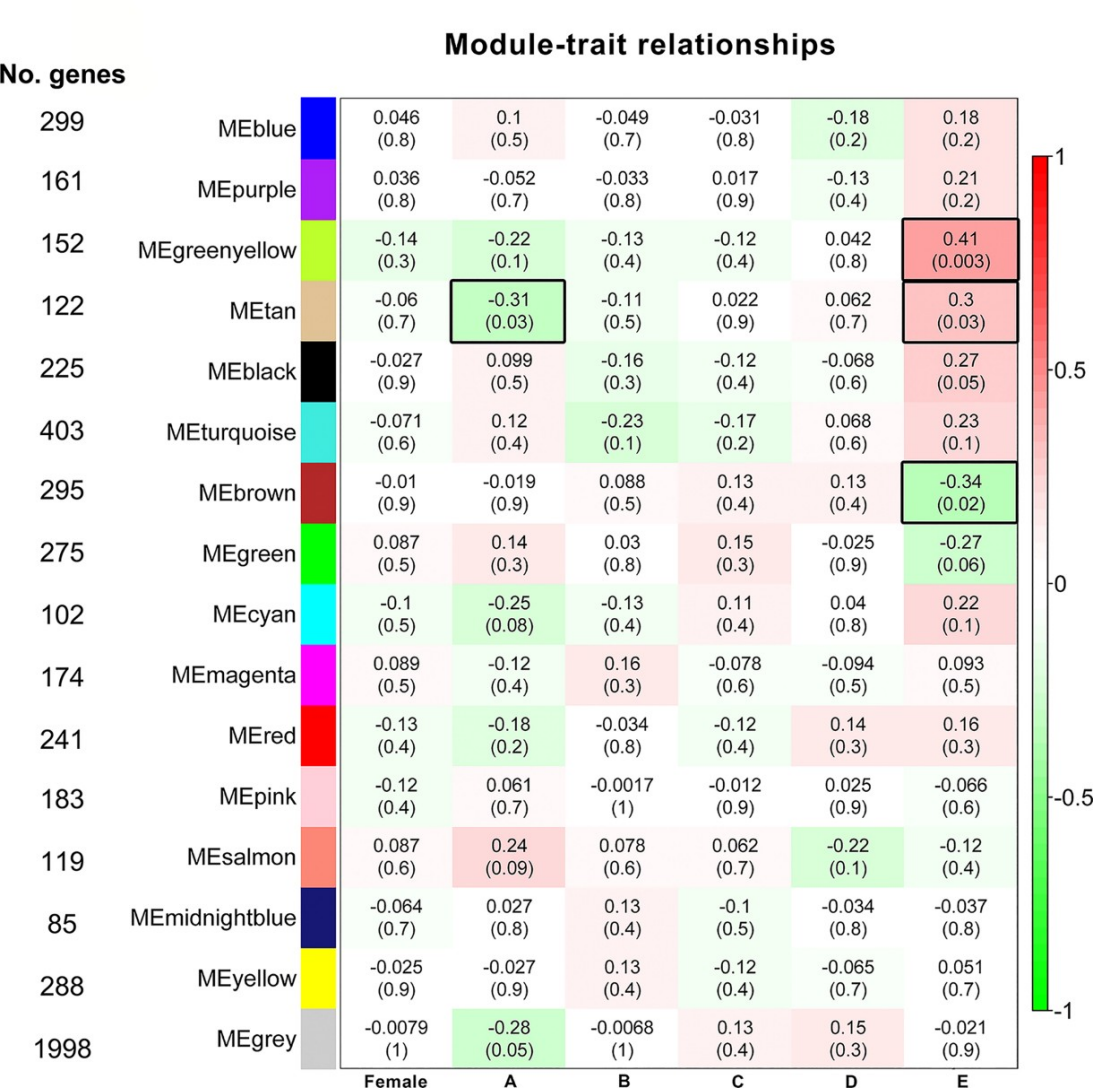
Module-trait relationships. WGCNA module significance (MS) correlations with gender and age groups. In the rows, module eigengenes (MEs) named by their module colors. In the columns, the traits of interest. Numbers inside each colored box are the correlation coefficients between the ME and the specific trait, with p-value in parentheses. The more intense the box color, the more negatively (green) or positively (red) correlated is the module with the trait (MS, as indicated by color bar). Black-border boxes highlight the significant module-trait relationships.

A gene co-expression network was constructed using the WGCNA package. After dynamic tree cut, the hierarchical clustering dendrogram identified 15 distinct gene modules, which contained from 85 (midnight blue module) to 403 genes (turquoise module). Genes not clustered in any module were grouped in the grey module. Subsequently, each age group was correlated with all the co-expression modules. This module-trait correlation analysis revealed three modules—tan, green yellow, and brown—that were significantly (p<0.05) associated with at least one age group (Fig 2). The green yellow module was positively correlated to group E (MS = 0.41, p = 0.003); the brown module was negatively correlated to group E (MS = -0.34, p = 0.02); while the tan module was negatively correlated to group A (MS = -0.31, p = 0.03), and it was positively and significantly correlated to group E (MS = 0.30, p = 0.03). None of the modules were significantly correlated with gender or with the age groups ranging from 31 days to 18 months (Fig 2).

#### Hub characterization

**Fig 3:**
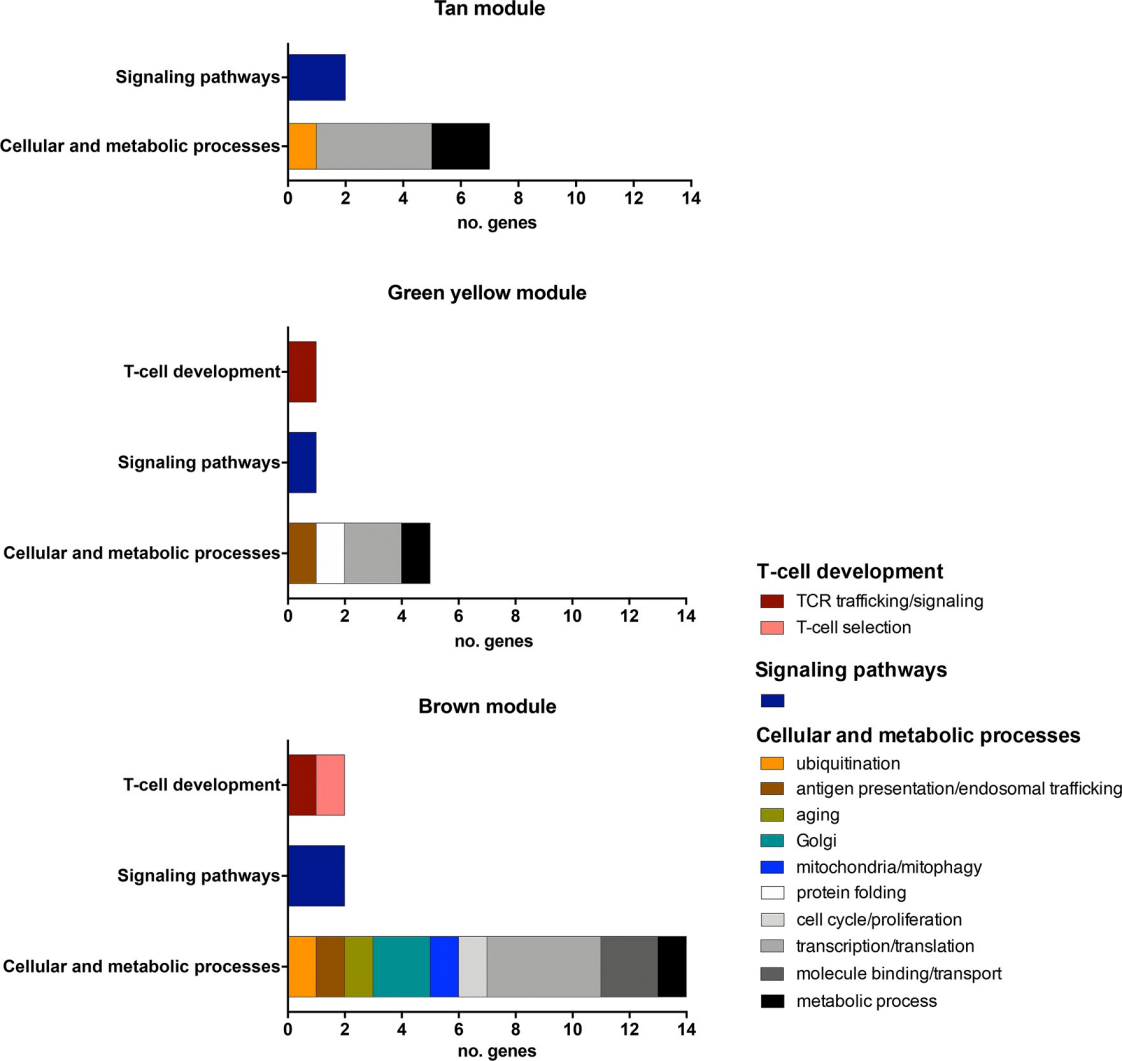
Hub classification according to functional categories. Biological functions assigned to all the 34 hubs distributed in three age-related modules: tan, green yellow, and brown.

Hierarchical categorization of genes in each significant module was accomplished through intramodular connectivity measures in order to identify the hubs, i.e. the network nodes presenting high *k*Within values (S2 Fig). Hubs correspond to the highly connected genes in a gene co-expression network[13]. A total of 34 hubs were thereby identified and their biological functions are presented in Table 1 and Fig 3 and detailed further ahead.

**Table 1:** Biological functions and TF-miRNA-hub gene co-expression correlations for the hubs found in modules highly associated with age groups.

			mRNA expression covariation	
Hub	Module	Biological function	miRNA-hub^a^	TF-hub (validated miRNA-TF)
***CAND1***	Tan	ubiquitination	*miR-181a-5p; miR-8069*	*ESR2*^e^ *(miR-150-5p)*
*ZNF675*	Tan	I-kappaB kinase/NF-kappaB signaling	*miR-4516*	
*ARHGEF15*	Tan	GTPase/signal transduction	*miR-181a-5p*	*POLR2A*^e^
*PQBP1*	Tan	transcription		
*ZNF430*	Tan	transcription	*miR-181a-5p*	
*ZNF493*	Tan	transcription		
*ZNF99*	Tan	transcription	*miR-4516*	
*LANCL3*	Tan	metabolic process		
*NAT8L*	Tan	metabolic process	*miR-181a-5p*	* *
***SNX17***	Green yellow	TCR trafficking		*POU5F1*^b^
*MTMR4*	Green yellow	endosomal trafficking		
*NKIRAS2*	Green yellow	I-kappaB kinase/NF-kappaB signaling		*POU5F1*^d^
*DNAJC4*	Green yellow	protein folding		*NANOG*^e^ *(miR-150-5p)*
*EDC3*	Green yellow	transcription		
*EIF3G*	Green yellow	translation		*NANOG*^e^ *(miR-150-5p); POU5F1*^b^
*COQ4*	Green yellow	coenzyme Q biosynthesis	*let-7a-5p*	* *
*PIK3CA*	Brown	TCR signaling	*miR-7641; miR-7975*	
*CHMP5*	Brown	T-cell positive selection	*miR-7975*	
*ARL8B*	Brown	antigen presentation		*SMAD4*^e^ *(miR-205-5p)*
*RNF138*	Brown	ubiquitination		
*NRIP1*	Brown	aging		
*TMEM167A*	Brown	Golgi-ER transport		*AHR*^c^ *(let-7a-5p; miR-181a-5p)*
*YIPF4*	Brown	Golgi		
*KRCC1*	Brown	mitochondria/mitophagy	*miR-7975*	
*RBM7*	Brown	cell cycle		*STAT4*^c^
*NMI*	Brown	STAT interactor	*miR-7975*	
*TMEM123*	Brown	cell surface receptor/signal transduction		
*OSBPL11*	Brown	lipid transport/molecular transport	*miR-7975*	
*SYPL1*	Brown	transport		
*CCDC59*	Brown	transcription		
*CNOT8*	Brown	transcription		*SMAD4*^b^ *(miR-205-5p)*
*HNRNPH2*	Brown	transcription		*HDAC2*^b^*; RAD21*^c^ *(let-7a-5p); SMAD4*^e^ *(miR-205-5p)*
*RBM34*	Brown	transcription		
GNG10	Brown	metabolic process	* *	* *

In bold, DE hubs that are also HGS genes. ^a^All miRNA-hub links have Pearson’s r = -0.5, except the link for *miR-181a-5p* –*NAT8L* which has r = -0.6. TF-hub links presented Pearson’s r = 0.6^b^ or 0.5^c^ or -0.6^d^ or -0.5^e^.

The tan module (negatively associated with group A and positively associated with group E) encompasses a total of nine hubs. Two of them—*CAND1* and *ZNF675* –are related to medullary thymic epithelial cells (mTECs). CAND1 is a putative autoimmune regulator (AIRE) targeted protein previously identified in mTECs[14] and as an AIRE interactor through gene-gene expression network analysis[4]. *ZNF675* (alias *TIZ*) codifies a negative modulator (zinc finger protein) of TRAF6[15], a signal transducer that activates the classical NF-κβ pathway playing a crucial role in mTEC development[16]. The other hubs in this module are related to transcriptional regulation (four genes), metabolic processes (two genes), and Rho GTPase signaling (one gene).

The green yellow module (positively associated with group E) has a total of seven hubs. Three of them are related to T-cell receptor (TCR) and thymic stromal functions. *SNX17* encodes a sorting nexin 17 that is involved in TCR trafficking and recycling[17]. *MTMR4* codes for myotubularin, a protein that localizes at the interface of early and recycling endosomes to regulate vesicular trafficking and maturation in the endocytic and autophagic pathways[18]. *NKIRAS2* acts as an inhibitor of NF-*κ*β activation[19] similarly to *ZNF675* (a hub in the tan module). The other four hubs in this module are related to transcriptional regulation, translation, protein folding, and metabolic process.

The brown module (negatively associated with group E) harbors a total of 18 hubs. Six of them are involved with T-cell development and antigen presentation-related functions, as follows. *CHMP5* enables positive selection by promoting post-selection thymocyte survival in part through stabilization of the pro-survival protein Bcl-2[20]. *PIK3CA* (aliases *PI3K*, *PI3kα*) codes for a phosphatidylinositol 3-kinase and acts in the regulation of T-cell receptor signal strength[21]. *ARL8B* localizes to MHC II compartments in dendritic cells and regulates the formation of MHC II-peptide complexes and antigen presentation[22]. *RNF138* is a member of the RING ubiquitin ligases subfamily structurally and functionally related to RNF125, or TRAC1, which exerts many roles in T-cell functioning, such as TCR recycling and activation of NF-*κ*β[23]. *NMI* is a STAT interactor and regulates the TGF-β/Smad pathway, a signaling pathway involved in the transcriptional regulation of *FOXP3*[24]. Interestingly, *NRIP1* codes for the nuclear receptor interactor protein 1 and it is involved in the regulation of aging processes[25]. The other hubs of this module are related to ER to Golgi transport, mitochondria, molecule transport, transcriptional regulation, signaling, cell cycle, and metabolic processes.

#### HGS gene characterization

The genes of the three modules significantly associated with age groups A (tan) and/or E (tan, green yellow, and brown) were also characterized according to module membership (MM) and gene significance (GS) values for groups A or E. Fifty genes with the highest GS values were selected (p<0.05) as high gene significance (HGS) genes (S3 Fig). Additionally, we conducted a gene expression comparative analysis between groups A and E (A vs E) for all genes of the three modules. A total of 134 genes (36 out of 122 in tan module, 60 out of 152 in green yellow module, and 38 out of 295 in brown module) are differentially expressed (DE) between groups A and E (Wilcoxon rank sum test, p<0.05).

Subsequently, we conducted a GO-based functional analysis for DE genes excluding all hubs and HGSs. The DE gene sets of each module were submitted to enrichment analysis using the Enrichr online web-based tool[26, 27] for identifying GO Biological Process (BP) terms. Only 18 (24%) DE genes could be classified in BP terms as relevant for thymus and/or immune system functioning (S2 Table).

**Fig 4:**
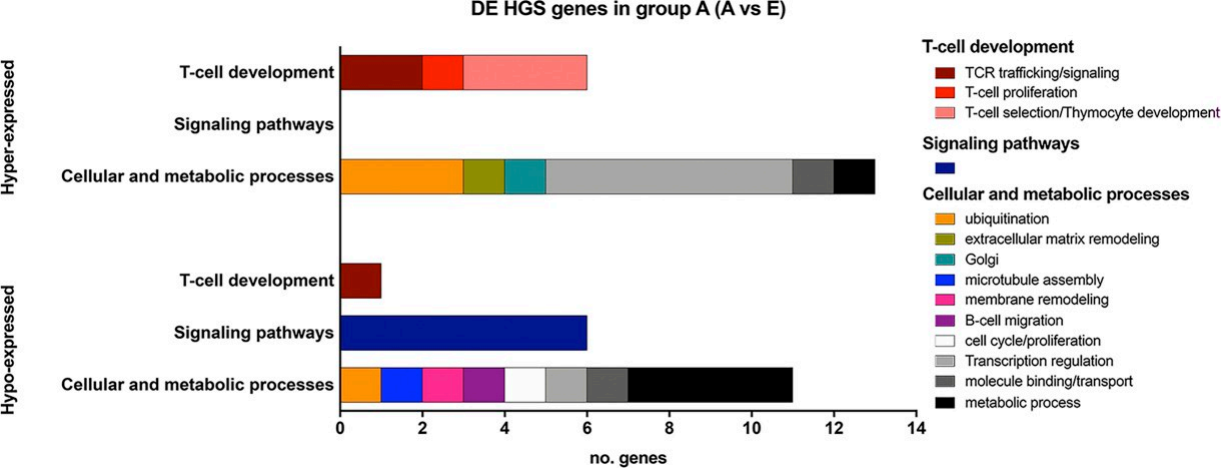
**Functional category classification for hyper- or hypo-expressed DE HGS genes in the group A (group A vs group E).** Biological functions were assigned to the 37 DE HGS genes distributed in the three age-related modules: tan, green yellow, and brown.

Among the 50 HGS genes, a set of 37 were found to be DE: 19 genes were hyper-expressed in group A and 18 in group E (Table 2 and Fig 4). Moreover, 34 of these DE genes varied significantly their expression across all age groups (Table 2). Among the hyper-expressed genes in group A, six are related to T-cell development and 13 to other cellular and metabolic processes. The hypo-expressed genes encompassed one gene related to T-cell development, six related to signaling pathways, and 11 related to other cellular and metabolic processes. The biological functions of these DE HGS genes are briefly commented ahead. It is interesting to mention that three of the hyper-expressed genes in the group A presented high fold-change values (>2.0): *CD5* and *CAND1*, in the tan module, and *SCML4*, in the green yellow module.

**Table 2:** Biological functions and TF-miRNA-HGS gene co-expression correlations for HGS genes found in modules significantly associated with the groups A and E.

	ANOVA	WT	FC			mRNA expression covariation	
HGS gene	adj-*p*	*p*		Module	Biological function	miRNA–HGS gene^a^	TF–HGS gene (miRNA)
**Hyper-expressed genes in group A**							
***PTBP1***	0.03	0.02	1.57	Tan	TCR signaling	*miR-181a-5p; miR-4516*	* *
*CD5*	0.00	0.00	2.06	Tan	TCR signaling		* *
CAND1*	0.04	0.02	2.49	Tan	ubiquitination	*miR-181a-5p; miR-8069*	*ESR2*^e^ *(miR-150-5p)*
***UBE3B***	0.01	0.00	1.62	Tan	ubiquitination	*miR-3960*	* *
*FAM126A*	ns	0.02	1.46	Tan	beta-catenin-TCF/Lef signaling pathway	***let-7b-5p***	* *
***SNRPA1***	0.02	0.01	1.37	Tan	RNA splicing		* *
*IFNAR2*	0.02	0.01	1.38	Green yellow	negative regulation of T-cell proliferation/IFN receptor		* *
*EIF2S1*	0.01	0.00	1.28	Green yellow	autophagy/T-cell selection		* *
*LOXL2*	0.00	0.00	1.84	Green yellow	extracellular matrix remodeling	*let-7b-5p*	* *
*SCML4*	0.00	0.00	2.38	Green yellow	transcription factor		* *
*C16orf53*	0.01	0.00	1.47	Green yellow	transcription		* *
*LLPH*	0.01	0.03	1.33	Green yellow	RNA binding		* *
*SULT4A1*	0.00	0.01	1.80	Green yellow	sulfotransferase/metabolic process	*miR-6869-5p*	* *
*SCLT1*	0.01	0.01	1.29	Brown	T-cell positive selection	*miR-205-5p*	* *
*CRBN*	0.01	0.02	1.29	Brown	ubiquitination	*miR-205-5p; miR-7975*	* *
*ATXN10*	0.02	0.01	1.29	Brown	Golgi dynamics and maintenance		*SMAD4*^e^ *(miR-205-5p)*
*PAPD4*	0.02	0.00	1.29	Brown	gene expression regulation/transcription regulation		* *
*ZCCHC6*	0.01	0.04	1.14	Brown	RNA binding/transcription regulation	*miR-205-5p*	*RARA*^c^
*C12orf23*	0.01	0.02	1.19	Brown	transmembrane protein/transport	*miR-7975*	* *
**Hypo-expressed genes in group A**							
***WNT5B***	0.01	0.00	-1.68	Tan	Wnt signaling pathway		* *
HSD3B7**	0.00	0.00	-1.83	Tan	B-cell migration		*POLR2A*^e^
TSPAN4**	0.01	0.02	-1.71	Tan	signal transduction/tetraspanin		*ESR2*^e^ *(miR-150-5p); POLR2A*^e^
***SIPA1L1***	ns	0.01	-1.37	Tan	GTPase activator/signaling pathways		*POLR2A*^c^
*PPAPDC1B*	0.00	0.00	-1.43	Tan	JAK-STAT signaling pathway		* *
***ZNF768***	0.01	0.00	-1.65	Tan	transcription regulation		*ESR2*^e^ *(miR-150-5p)*
***CALHM2***	0.01	0.00	-1.54	Tan	calcium homeostasis modulator/molecule transport		*ESR2*^c^ *(miR-150-5p)*
*NLE1*	0.03	0.01	-1.33	Tan	ribosome biogenesis/metabolic process		* *
SNX17*	0.04	0.03	-1.30	Green yellow	TCR trafficking		*POU5F1*^b^
*TUBA1A*	0.01	0.02	-1.36	Green yellow	microtubule assembly		* *
*CC2D1B*	0.00	0.01	-1.29	Green yellow	membrane remodeling		
*CNTROB*	0.02	0.01	-1.48	Green yellow	cell proliferation		*JUN*^e^ *(miR-342-3p); NANOG*^c^ *(miR-150-5p)*
*MAP1A*	0.01	0.01	-1.40	Green yellow	microtubule assembly/signaling pathways		* *
*ILVBL*	0.01	0.02	-1.46	Green yellow	protein binding/metabolic process		* *
*FAHD2A*	0.00	0.01	-1.36	Green yellow	metabolic process		* *
*TARBP2*	ns	0.02	-1.24	Green yellow	RNA binding process/metabolic process		* *
*UBAP2*	0.04	0.02	-1.23	Brown	ubiquitination		* *
*PRR11*	0.01	0.02	-1.53	Brown	Wnt/beta-catenin signaling pathway		* *

In bold, genes that are HGS genes only in group A; WT: Wilcoxon rank sum test (AxE), FC: Fold change, calculated as the ratio between the mean relative expression for groups A and E (A/E); *HGS genes that are also hubs; **HGS genes in both groups A and E; ns: no significant. ^a^All miRNA-HGS gene links have Pearson's r = -0.5. TF-hub links presented Pearson’s r = 0.6^b^ or 0.5^c^ or -0.6^d^ or -0.5^e^.

In age group A, 10 HGS genes were identified in the tan module, the only module associated with this group (genes in bold, Table 2). Two of these genes, *WNT5B* and *PTBP1*, are related to thymocyte development. *WNT5B* is a member of the WNT gene family and codes for a signaling glycoprotein. In mice, Wnt glycoproteins regulate *Foxn1* transcription in TECs, therefore providing regulatory signals critical for thymic function. Wnt5b transcripts are present in thymocytes, peripheral T-cells and epithelial cells. Their expression is maximal in DP thymocytes, decreasing by 75–95% at later stages of T-cell development[28]. *PTBP1* codifies for an RNA binding protein that plays a critical role in early T lymphocyte ontogeny by regulating the expression of CD5 and, consequently, TCR signaling[29]. Two other genes—*CAND1* and *UBE3B* –are primarily related to ubiquitination and involved with mTECs and tolerance induction. *CAND1*, also a hub gene, codifies for a Cullin-RING ubiquitin ligase (Table 1) and, as mentioned before, is a putative AIRE-targeted protein previously identified in mTECs[4, 14]. The gene *UBE3B* codes for a HECT (homology to E6-AP C-terminus) domain-containing E3 ubiquitin ligase. HECT ligases have been linked to the induction of immune self-tolerance[30]. Lastly, *HSD3B7* (also an HGS gene in group E) is related to B-cell migration in lymphoid tissues[31], but its function in thymic tissue is hitherto undescribed. The remaining five HGS genes in age group A are related to other cellular processes (Table 2).

In the age group E, we identified 29 HGS genes (Table 2): six in the tan module (of which two are also HGS genes in group A), 15 in the green yellow module, and 8 in the brown module. Five of these 29 HGS genes are related to T-cell development. Two of them—*CD5* and *SNX17*—are involved in TCR trafficking/signaling. CD5 (tan module) codifies for a glycoprotein found on thymocyte’s membrane and it is expressed early in T lymphocyte ontogeny functioning as negative regulator of TCR-mediated signaling, fine-tuning the TCR signaling response during thymocyte selection[32]. It is worth to note that *CD5* expression is regulated by *PTBP1*[29], an HGS gene in group A. *SNX17* (green yellow module), also a hub gene, is involved in TCR trafficking and recycling[17]. *SCLT1* (alias *CAP-1A*) codifies for a clathrin adaptor and is related to T-cell positive selection. Inhibition of *CAP-1A* expression in thymocytes blocks progression from double-positive immature thymocytes, resulting in complete absence of SP CD4+ thymocytes and severe reduction of SP CD8+ thymocytes[33]. *IFNAR2*, codes for one chain of a receptor for interferons alpha and beta and is related to T-cell proliferation, acting in the interferon signaling pathway. In mice, it was shown that a gain-of-function mutation in this receptor inhibits interferon alpha and beta signaling in thymocytes, transducing an antiproliferative response[34]. *EIF2S1* is related to autophagy and thymocyte selection. *EIF2S1* codifies a translation initiation factor that promotes the expression of *ATG12* when phosphorylated[35]. *ATG12* codes for a ubiquitin-like molecule that acts as a core autophagy protein to control late endosome function and has a crucial role in thymocyte development and selection[36].

Four HGS genes—*MAP1A*, *PPAPDC1B*, *PRR11*, and *FAM126A* –are involved in signaling pathways. *PPAPDC1B* (alias *PLPP5*), a phospholipid phosphatase 5, is involved in the JAK-STAT signaling pathway[37]. *PRR11* codifies a proline-rich protein that activates the Wnt/β-catenin signaling pathway[38]. *MAP1A* belongs to the microtubule-associated protein family. It modulates microtubule function and assembly and, through the interaction with Rho GTPases, facilitates vesicle trafficking and regulates the trafficking of signaling molecules[39]. *FAM126A* is a gene involved in the β-catenin-TCF/LEF signaling pathway[40], whose members are expressed in thymocytes and play a role in T-cell development[41].

Two other HGS genes–*CRBN* and *UBAP2* –are involved in ubiquitination. *CRBN*, which is highly expressed in CD4+ T-cells, forms a complex with E3 ubiquitin ligase and regulates sensitivity to TCR stimulation[42]. *UBAP2* codifies an ubiquitin associated domain-containing protein (UBA) acting in the ubiquitin-dependent proteasomal degradation[43], a pathway involved in relevant thymic functions, such as the generation of MHC class I ligands[44].

The remaining 16 HGS genes in the group E (not including *HSD3B7*, commented before, and *TSPAN4*, which are HSG genes in A and E groups) are related to a variety of cellular and molecular processes in the thymic tissue (Table 2). It is interesting to note that one of these genes, *LOXL2*, plays a key role in extracellular matrix remodeling[45] and is highly expressed in thymus[46].

### Abundantly and differentially expressed miRNAs

Thymic tissue global miRNA expression data from 29 patients (S3 Table) were used for identifying abundantly expressed miRNAs and for comparative analysis within the five A-E sequential age intervals (see Methods). We found that 19 out of 428 miRNAs were abundantly expressed (about 40-times average increase) in at least one age group, and that nine of these miRNAs were abundantly expressed in all age groups (S3 Table). Additionally, through ANOVA comparative analysis we identified two miRNAs–*let-7b-5p* and *miR-205-5p* –as being differentially expressed across age groups (DE, p<0.05). Moreover, these two DE miRNAs were abundantly expressed in all age groups except in the group B (31 days-old to 6 months-old). On the other hand, four miRNAs—*miR-25-3p*, *miR-494-3p*, *miR-6087*, and *miR-7641*—are abundantly expressed only in the group B. It is interesting to note that other three miRNAs—*miR-181a-5p*, *miR-16-5p*, and *let-7a-5p* - are abundantly expressed in all groups except group E (19 to 31 months-old).

### Search for transcription factors covarying with hubs and HGS genes

The sets of hubs and HGS genes were submitted to enrichment analysis using the Enrichr online web-based tool[26, 27] for identifying protein-protein interactions with TFs (Transcription Factor–PPIs Database). A total of 63 TFs were found as interactors of hubs and HGS genes (p<0.05). However, we selected only the TFs that were expressed in our global mRNA expression data set: 14 interacted only with hubs and 27 only with HGS genes, while 28 TFs were found to be common to hubs and HGS genes (S4 Table). Additionally, all these 63 TFs were also used for searching TF-miRNA interactions using the miRTarBase Database. A total of 1,195 miRNAs were found. Subsequently, we selected TF-miRNA interactions involving only the abundantly expressed miRNA detected in this study. A total of 11 out of 19 abundantly expressed miRNAs were found interacting with TFs (S5 Table).

### Hub/HGS-miRNA-TF co-expression correlations

**Fig 5:**
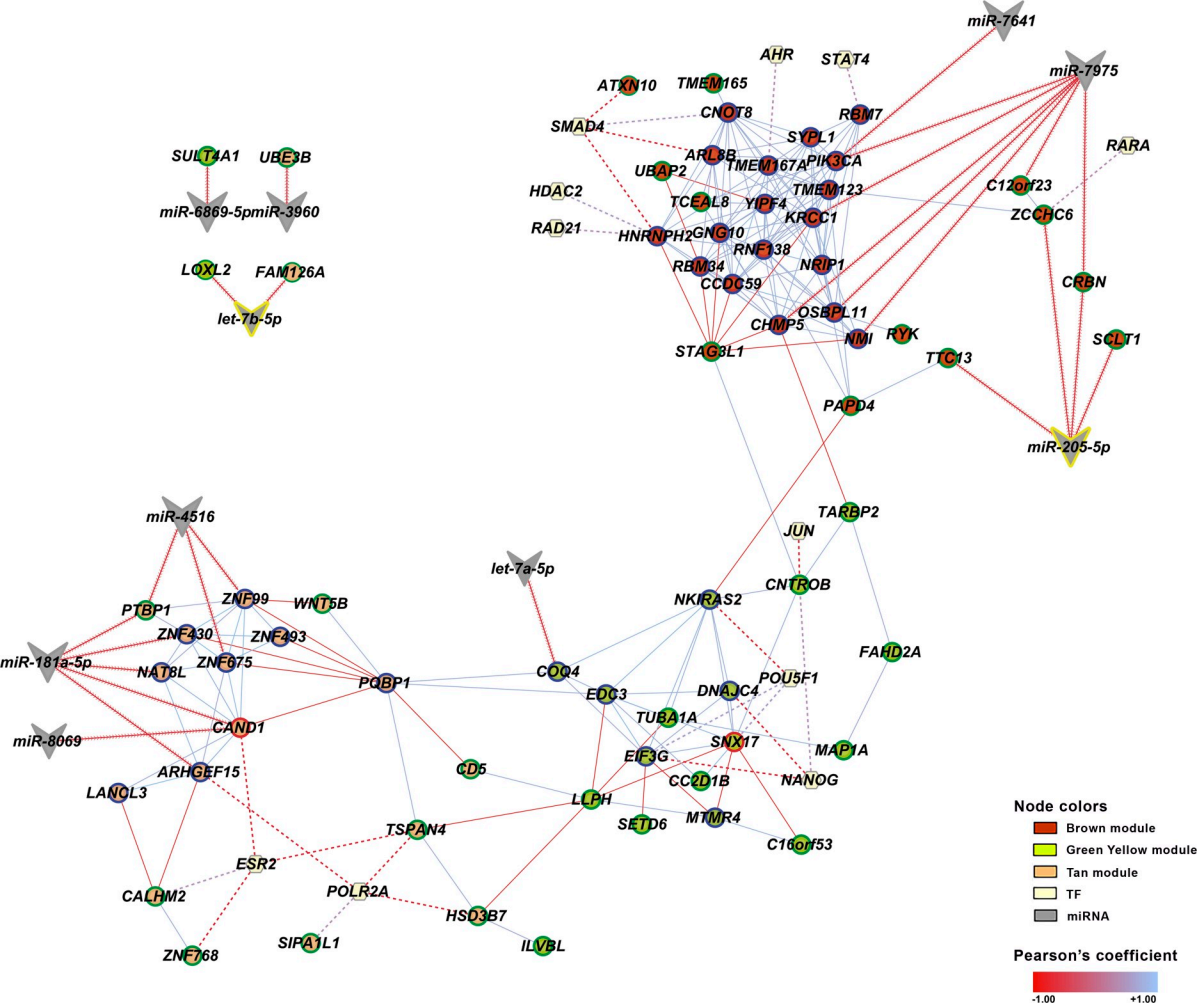
Integrative TF-miRNA-mRNA co-expression subnetwork of the abundantly expressed miRNAs covarying with hubs, HGS genes, and transcription factors (TFs). Nodes corresponding to hubs or HGS genes are depicted by their respective module color. Only co-expression covariance values of ≥ |0.70| between gene-gene (solid lines), ≤ -0.50 between gene-miRNA (arrowed lines), and ≥ |0.50| gene-TFs (dashed lines) were considered. Positive (blue) and negative (red) co-expression interactions are depicted by a color gradient based on Pearson’s coefficient values. Abundantly expressed miRNAs are depicted by vees; abundantly expressed and DE miRNAs are highlighted with a yellow border; HGS genes are depicted by green border nodes; hubs are depicted by blue border nodes; the two HGS genes that are also a hub gene are depicted by red border nodes. TFs are depicted by hexagons.

The co-expression correlation analysis integrating mRNA expression data of hubs and HGS genes, the 19 abundantly expressed miRNAs and the 63 selected TFs was accomplished by Pearson´s correlation (Tables 1 and 2). This integrative analysis allowed the construction of a co-expression network showing all the hub/HGS-miRNA-TF interactions (Fig 5). A total of 11 hubs (14 miRNA-hub interactions) and 13 HGS genes (13 miRNA-HGS gene interactions) presented high co-expression interactions (*r* ≤ -0.50) with at least one miRNA. Ten miRNAs showed correlations with hubs or HGS genes. One miRNA–*let-7a-5p –*interacted with just one hub, while four miRNAs–*let-7b-5p* (DE), *miR-205-5p* (DE), *miR-3960*, and *miR-6869-5p –*had links only with HGS genes. Other five miRNAs—*miR-181a-5p*, *miR-4516*, *miR-7641*, *miR-7975*, and *miR-8069 –*had links with hubs and HGS genes as well (Tables 1 and 2, Fig 5).

It is interesting to note that nine out of the ten miRNAs linked with hubs or HGS genes appear to be module-specific. Four out of those nine miRNAs—*miR-181a-5p*, *miR-4516*, *miR-8069*, and *miR-3960* –covaried only with genes belonging to the tan module. Other three miRNAs—*miR-7641*, *miR-7975*, and *miR-205-5p* –covaried only with brown module genes. Finally, two miRNAs—*let-7a-5p* and *miR-6869-5p* –covaried with genes in the green yellow module. So far, only the interaction between *let-7b-5p* and *FAM126A* (an HGS gene in the tan module) has been experimentally validated in human tissues (miRTarBase).

A total of 11 out of 63 TFs presented high gene co-expression interaction values (*r* ≥|0.50|) with hubs (14 TF-hub interactions) and with HGS genes (12 TF-HGS gene interactions). Seven out of these 11 TFs presented validated interactions (miRTarBase) with at least one abundantly expressed miRNA (Tables 1 and 2). The brown module encompasses the majority of these TFs: four of them—*STAT4*, *AHR*, *HDAC2*, and *RAD21*—are correlated just with hubs and two–*SMAD4* and *RARA–*are correlated with hubs and HGS genes. In the green yellow module two TFs—*POU5F1* and *NANOG—*are linked with hubs and HGS genes, and another, *JUN*, is linked to just one HGS gene. In tan module two TFs—*ESR2* and *POLR2A* –are correlated with hubs and HGS genes (Tables 1 and 2, Fig 5). The literatures describe the majority of these TFs as playing important roles in thymus functioning and/or T-cell development, excepting *ESR2* and *POLR2A*. However, it should be noted here that *POLR2A* is an *AIRE* interactor[4, 14] and that *ESR2* (estrogen receptor 2) interacts with *CAND1* (a hub and HGS gene), whose interaction with *AIRE* is modulated by sex steroids[4].

An integrative TF-miRNA-mRNA co-expression network of the abundantly expressed miRNAs covarying with hubs, HGS genes, and transcription factors was subsequently constructed (Fig 5). It shows that the majority of the hub-hub or HGS-HGS gene links have positive correlations, while many hub-HGS gene links present negative correlations. Moreover, there are more hub-hub links than hub-HGS genes or HGS-HGS links. This result indicates that hubs are related to network module robustness and the HGS genes–which are differentially expressed genes–are either bridges between modules or border genes.

## Discussion

The three age-related transcriptional modules here identified are correlated with two distinct and characteristic time intervals in human thymic evolution during the first 2½ years of life: the neonatal period (age group A) and the fourth/fifth semester period (age group E). In the neonatal period a transient thymic involution takes place, marked by severe cortical DP thymocyte depletion and pronounced changes in the extracellular matrix network[3], whereas in the fourth semester of life the thymic involution becomes histomorphologically patent through the initial decline of the total amount of lymphatic tissue[1]. Interestingly, no transcriptional module was correlated with any interval in the 31 days-18 months period, along which the thymus reaches its maximal growth[1]. Our TMA-IHC data on thymic cell subpopulations reflects this scenario, showing a continuous and moderate increase of thymocyte and B-cell numbers (S1 Fig). These findings indicate that a genomic mechanism may act, synergistically with physiological and environmental stimuli[4, 5], on early thymic evolution/involution programming.

Hub and HGS genes were hierarchically identified and functionally characterized for the three age-related modules. Almost all hubs (32 out of 34) are non-DE genes and related to cellular and metabolic processes, with just a few (3 out of 34) involved in T-cell development (Table 1 and Fig 3). Hubs are nodes with high number of interactions with other nodes in a transcriptional module, occupying a topologically central position in the module and conferring robustness to the network[13, 47]. Thus, it is expectable that their expression profiles remain quite constant, since hubs are rarely causal to phenotypes and usually dispensable for growth or adaptation processes[13].

Inversely, most of the HGS genes are DE genes between A and E age groups and most of these genes presented significantly variation across all age groups (Table 2). HGS genes frequently are tissue specific, causal to phenotypes (including disease phenotypes), and tend to be located at the periphery of gene co-expression networks[48], as shown here in Fig 5. HGS genes are thus biologically significant for particular traits[12], such as age in thymic development. Indeed, one third of the hyper-expressed HGS genes in the age group A (neonate) were related to T-cell development, against one-twentieth in age group E. These differences (Fig 4) may correlate with the severe depletion of DP T-cells in the neonatal thymus, which is followed by a subsequent recovery of thymic T-cell populations at the end of the first month of life, very well characterized by Varas et al.[3].

As mentioned above, the expression profiles for abundantly expressed miRNAs show some differences among the age groups (S3 Table). Four miRNAs are abundantly expressed just in the group B, the only group where *let-7b-5p* and *miR-205-5p* are not abundantly expressed. The age group E is the only one where *let-7a-5p*, *miR-16-5p* and *miR-181a-5p* are not abundantly expressed. Interestingly, group B corresponds to the period of minipuberty[49], where sex steroid hormones affect thymic functioning[4], and group E corresponds to the period where thymic involution begins[1]. In addition, some of these abundantly expressed miRNAs were previously described as involved in relevant thymic functions. *MiR-181a-5p* expression is required for positive and negative thymocyte selection[50]; it was shown to regulate mTEC proliferation and age-related thymic involution in mice[51]. *MiR-205-5p* is highly expressed in mTECs and its expression changes correlate with thymic development and involution[52]. *MiR-150-5p*, which is abundantly expressed in all five age groups, is important for T-cell maturation, being upregulated after the DP stage[53].

The TF-miRNA-Hub/HGS co-expression correlations, depicted in Tables 1 and 2 and in Fig 5, help to compose the genomic scenario of the human early thymic evolution. Firstly, it is clear that the three age-related modules and their respective hubs are regulated by different and quite specific sets of abundantly expressed miRNAs and TF-hub interactions (Table 1). The same situation prevails for the HGS genes, though it should be noted that just three TFs and several abundant miRNAs interact with the hyper-expressed genes in the age group A, whereas six different TFs but no abundantly expressed miRNA interact with the hypo-expressed genes in this group (Table 2). The validated TF-miRNA interactions occurred more frequently with *miR-150-5p*, *miR-181a-5p* and *miR-205-5p*, already mentioned as relevant for thymic functions.

Altogether, our results show a genomic mechanism differentially governing the cellular and molecular processes involved in the functioning of the neonate thymus and, later on, in the beginning of thymic decline. Along the first two years of age, this mechanism is tightly regulated by the differential expression of HGS genes and by TF-miRNA-hub/HGS interactions.

## Material and methods

### Patients and thymic tissue specimens

Thymic tissue samples were obtained from 57 karyotypically normal patients who underwent cardiac surgery at Instituto Dante Pazzanese de Cardiologia, São Paulo, Brazil. Patient’s demographic data are presented in S1 Table. The samples were grouped by age intervals: 0–30 days (A), 31days-6 months (B), 7–12 months (C), 13–18 months (D), and 19-31months (E). Fresh corticomedullar sections of thymic tissue were obtained at surgery room and immediately preserved with RNA*later* (Qiagen, cat. no. 76106). The research ethics committee of Instituto Dante Pazzanese de Cardiologia has approved this research under number 4287. All methods were performed in accordance with the relevant guidelines and regulations. Written informed consents have been obtained from parents and/or legal guardians.

### Tissue microarray and immunohistochemistry techniques (TMA-IHC)

The specimens were fixed in formalin for 24h and embedded in paraffin. TMA was prepared according procedures using a precision mechanized system (Beecher Instruments, Silver Springs, MD, USA). Three samples of each, cortical and medullary areas, were represented for each case. Sections (4μm) cut from tissue microarrays (TMAs) blocks were used for immunohistochemistry. These sections were stained for the expression of cytokeratin AE1/AE3 (DAKO, AE1/AE3, 1:800), CD4 (LEICA NOVOCASTRA, 4B12, 1:50), CD8 (DAKO, C8/144B, 1:400), and CD20 (DAKO, L26, 1:2500) in order to visualize epithelial cells, and T- and B-cells in thymus. The final reaction product was visualized in brown with diaminobenzidine as chromogen. Cell nuclei were stained with Harris’s hematoxylin. Histological examination was performed using an Olympus CX31 microscope and images were captured with a Canon EOS Rebel SL1 digital camera. The images were analyzed using the Image-Pro Plus program, version 5 (Media Cybernetics). Cells were counted in three TMA areas of interest for each patient’s sample. For every sample, the proportion of cells in each cellular subpopulation was calculated using the average area values of the three areas of interest, and for the average amount of cells in the three areas. Correlation data analysis (Pearson’s r) was used for evaluating the amount of cells distributed on cortical and medullary areas (in percentage) along the age intervals (in months).

### Total RNA extraction

Thymus tissue explants (3–4 mm^3^) were extracted using TissueRuptor and RNeasy Lipid Tissue Kit (Qiagen). RNA quality was assessed on the Agilent BioAnalyzer 2100 (Agilent Technologies) and stored at -80°C.

### Microarray hybridization

In order to determine gene expression profiles, 4x44K v.2 DNA microarrays (Whole Human Genome Microarray Kit, G4845A, Agilent Technologies) were used. The procedures for hybridization using the fluorescent dye Cy3 followed the manufacturer’s protocols (One-Color Microarray-Based Gene Expression Analysis–Low Input Quick Amp Labeling and miRNA Complete Labeling and Hyb Kit, Agilent Technologies). For miRNA, whole human miRNA of 8x60K DNA microarrays (Human miRNA Microarray slide, G4872A, Agilent Technologies), containing probes for 2,549 human miRNAs based on miRBase database (release 21.0) were used.

The images were captured by the reader Agilent Bundle according to the parameters recommended for bioarrays and extracted by Agilent Feature Extraction software version 9.5.3 for gene expression and 10.7.3 for miRNA expression. Spots with two or more flags (low intensity, saturation, controls, etc.) were considered as NA, that is, without valid expression value. An in-house algorithm in R environment (version 3.4.4) was used for: i) sample grouping; ii) excluding the mRNA transcript spots presenting at least one NA per group or the miRNA transcript spots presenting more than 50% of NAs in the group; iii) converting gene expression values to log base 2. Data normalization was performed using limma package in R environment (version 3.4.4).

All mRNA and miRNA microarray raw data have been deposited in GEO public database (http://www.ncbi.nlm.nih.gov/geo), a MIAME compliant database, under accession number GSE131242 (Reference Series).

### Weighted Gene Co-expression Network Analysis (WGCNA)

The WGCNA package is a comprehensive collection of R functions for performing various aspects of weighted correlation network analysis[54]. Through WGCNA it is possible to identify and characterize gene modules whose members share strong co-expression[12, 55]. A total of 5,344 GO annotated genes were used for WGCNA across 50 samples (S1 Table).

Pearson’s correlation coefficient was used for obtaining gene co-expression similarity measures and for the subsequent construction of an adjacency matrix using soft power and topological overlap matrix (TOM). Soft-thresholding process transforms the correlation matrix to mimic the scale free topology. Module identification is based on TOM and in average linkage hierarchical clustering. Keeping to the scale-free topology criterion, soft power β = 9 (R^2^ = 0.87) was considered (S4 Fig). Finally, dynamic cut-tree algorithm was used for dendrogram’s branch selection. The module eigengene (ME) is defined as the first principal component of a given module, which can be considered a representative of the gene expression profiles in a module. Module Membership (MM), also known as eigengene-based connectivity (*k*ME), is defined as the correlation of each gene expression profile with the module eigengene of a given module[12].

#### Module-trait association

We obtained the gene significance (GS) value of the correlation between the gene expression and the traits (here represented by gender and age intervals: groups A—E). The mean GS considered for a module is the measure of the module significance (MS). The GS values were obtained using Pearson’s correlation and Student’s t-test was used to assign a p-value to the module significance. The modules presenting a significant p-value (p<0.05) were selected for biological functional analysis.

#### Intramodular analysis for hub selection

Modules significantly correlated to one or more traits were deeply evaluated for identifying relevant hubs, i.e. genes presenting high connectivity values related to the network (overall connectivity) and to the module (intramodular connectivity for each gene based on its Pearson’s correlation with all other genes in the module), determined by a *k*Total (x-axis) *vs k*Within (y-axis) scatter-plot.

#### Identification of HGS genes

Modules that showed high correlation with one or more age intervals were selected for the identification of genes presenting high GS values (HGS genes), determined by a MM (x-axis) *vs* GS (y-axis) scatter-plot. Differential gene expression analysis between groups A and E was performed by Wilcoxon Rank Sum Test and the comparison across all five age groups was accomplished by ANOVA. We considered p<0.05 as significant for both analyses. Subsequently, fold-change was calculated by the mean relative expression ratio of groups A and E.

#### Functional enrichment analysis for selected module genes

Gene sets of those significant trait-associated modules were submitted to enrichment analyses, using the Enrichr online web-based tool[46], to identify significantly over-represented terms on GO Biological Process, (S6 Table), Transcription Factor–PPIs Database, and miRTarBase (S4 and S5 Tables) among the gene annotations. Moreover, we applied the same enrichment analysis’ strategy for all hubs and HGS genes.

### miRNA analysis

The abundantly expressed miRNAs for the five age intervals were selected after analyzing miRNA expression values distribution through a scatter dot plot[4]. Cut-off values closer to the inflection point (S5 Fig.) were adopted, as follows: 453, 433, 610, 514, and 685 for A, B, C, D, and E groups, respectively. ANOVA were used for identification of the differentially expressed miRNAs and p < 0.01 was considered significant (S3 Table).

### mRNA-miRNA-TF integrative subnetworks analysis

Gene-miRNA co-expression covariance was obtained by Pearson’s correlation (*r* coefficient) for all 34 hubs and 37 DE HGS genes, and the abundantly expressed miRNAs. The same co-expression covariance analysis was obtained for all regulating transcription factors (TFs) related to any hub and/or HGS gene—according to the Transcription Factor–PPIs Database–that also presented significant (p<0.05) validated interactions (miRTarBase) with the abundantly expressed miRNAs (S4 and S5 Tables).

Thus, a gene-miRNA integrative subnetwork was constructed for the abundantly expressed miRNAs and the hubs/HGS genes/TFs, considering only co-expression correlation values ≥|0.70| for gene-gene (positive and negative co-expression interactions) and ≥|0.50| for gene-miRNA (only negative co-expression interactions) and for gene-TF interactions. The absolute r values distribution and the inflection points for gene-gene, miRNA-gene, and TF-gene links appear in S6 Fig.

## Supporting information

S1 Fig Distribution of CD4+, CD8+, CD20+, and AE1/AE3+ cells in the thymus cortex and medulla assessed by TMA-IHC. Correlation data analysis (Pearson’s r) was used for evaluating the number of cells distributed on cortical and medullary areas (in percentage) along the age intervals (in months). Significant correlations were observed for medullary CD4+ (r = 0.469, p = 0.034) and CD20+ (r = 0.714, p = 0.001) stained cell areas, and for cortical CD8+ (r = 0.462, p = 0.036) stained cell area. No significant correlation was seen for cells stained for AE1/AE3. A linear regression trend line was drawn for cortical and medullary area measurements.
(TIF)

S2 Fig Intramodular hub selection. *k*Total vs *k*Within plots for modules tan (**a**), green yellow (**b**), and brown (**c**). Hubs are identified by colored dots and their respective gene symbols.
(TIF)

S3 Fig Intramodular high GS (HGS) gene selection for groups A (0–30 days) and E (19–31 months). Module Membership (MM) vs. Gene Significance (GS) plots for the module tan (**a-b**)–groups A and E–and the modules green yellow (**c**) and brown (**d**)–group E. Hyperexpressed and hypoexpressed genes for a given trait present positive and negative GS values, respectively. HGS genes are identified by colored dots and their respective gene symbols.
(TIF)

S4 Fig Selection of the soft-thresholding power (β). The dataset was fit to a scale-free model of proposed values for β ranging from 1 to 35 (numbers inside the plots). Approximate scale-free topology is attained around soft-thresholding power of 9, which reflects the inflection point where model fit begins to decrease with power increasing (left panel). The plot of the mean connectivity along with the soft-thresholding power (right panel). The red line indicates the scale-free topology R2 fit index cut-off of 0.8700.
(TIF)

S5 Fig Distribution of miRNAs’ expression in the five age groups. The red dashed lines indicate the cut-off values for selecting abundantly expressed miRNAs for each of the five age groups.
(TIF)

S6 Fig Absolute r values distribution. The red dashed lines indicate the cut-off values for selecting link thresholds for gene-gene, miRNA-gene, and TF-gene interactions.
(TIF)

S1 Table Patient`s demographic data from 57 samples included in WGCNA and/or miRNA and TMA-IHC analyses. (XLSX)

S2 Table GO Biological Process search (Enrichr) for DE genes of the modules tan, green yellow, and brown. Terms highlighted in grey correspond to thymus and/or immune system functioning.
(XLSX)

S3 Table Abundantly and differentially expressed miRNAs. (XLSX)

S4 Table Transcription Factor–PPIs Database search (Enrichr) for regulatory transcription factors (TFs) described to hubs/HGS genes. (XLSX)

S5 Table MirTarBase search (Enrichr) for validated interactions described for the 19 abundantly expressed miRNAs and the 63 transcription factors (TFs). (XLSX)

S6 Table Functional characterization of modules' genes according to GO Biological Process. (XLSX)
