# Correction: Age-related transcriptional modules and TF-miRNA-mRNA interactions in neonatal and infant human thymus

**DOI:** 10.1371/journal.pone.0235767

**Published:** 2020-07-01

**Authors:** The PLOS ONE staff

The Funding statement is incorrect. The correction Funding statement is as follows: This work was funded by Fundação de Amparo à Pesquisa do Estado de São Paulo (FAPESP) research grants 2015/22308-2 (to CAM-F) and 2014/50489-9 (to MMC-S) and Conselho Nacional de Desenvolvimento Científico e Tecnológico (CNPq) grant306893/2018-5 (to CAM-F). The funders had no role in study design, data collectionand analysis, decision to publish, or preparation of the manuscript.

The publisher apologizes for the error.
