# Supplementary figures and images for: Age-related transcriptional modules and TF-miRNA-mRNA interactions in neonatal and infant human thymus
Source: PLoS One. 2020 Apr 15;15(4):e0227547. doi: 10.1371/journal.pone.0227547 (PMC7159188; doi:10.1371/journal.pone.0227547)

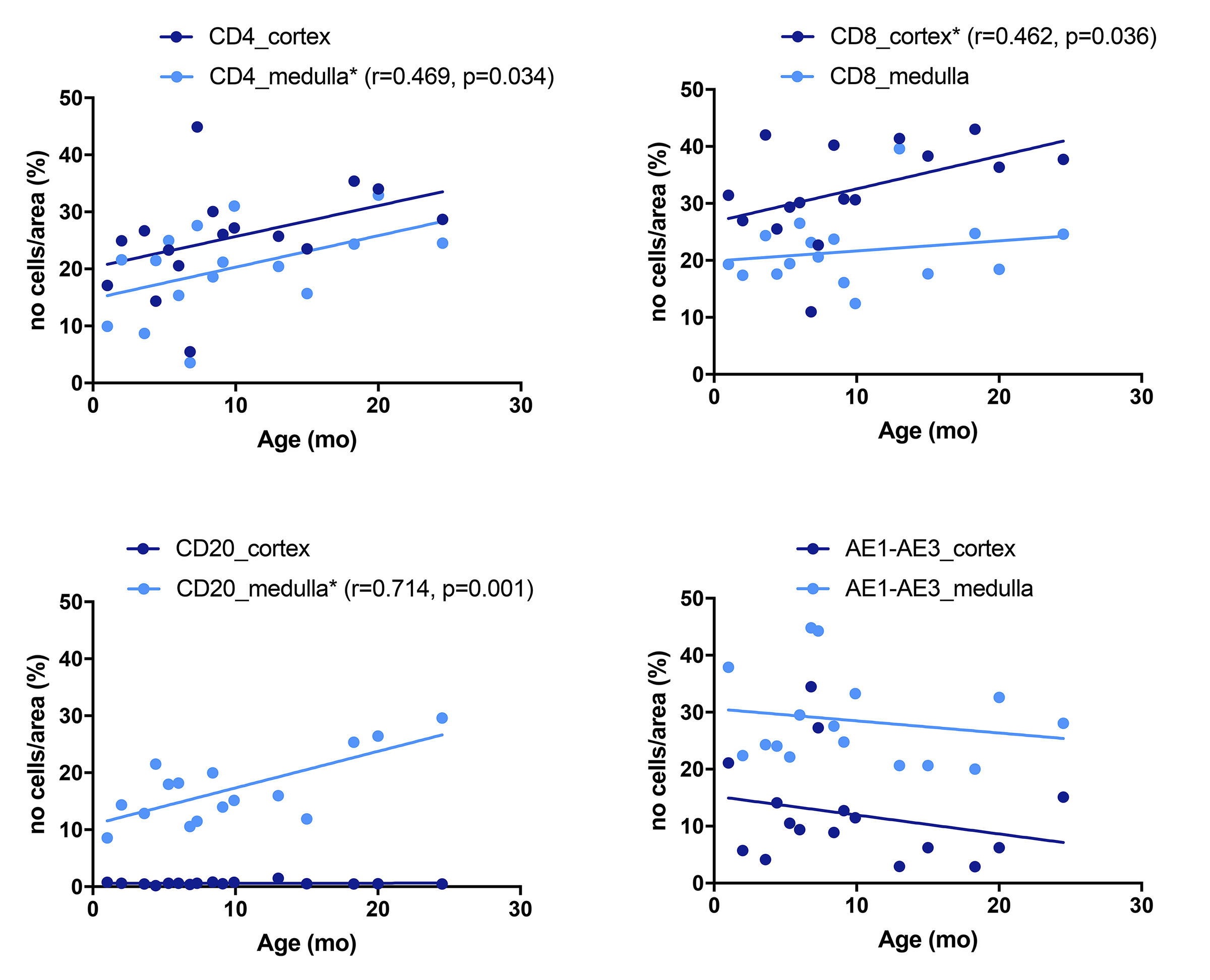

Supplement: S1 Fig — Correlation data analysis (Pearson’s r) was used for evaluating the number of cells distributed on cortical and medullary areas (in percentage) along the age intervals (in months). Significant correlations were observed for medullary CD4+ (r = 0.469, p = 0.034) and CD20+ (r = 0.714, p = 0.001) stained cell areas, and for cortical CD8+ (r = 0.462, p = 0.036) stained cell area. No significant correlation was seen for cells stained for AE1/AE3. A linear regression trend line was drawn for cortical and medullary area measurements. (TIF) [file pone.0227547.s001.tif]

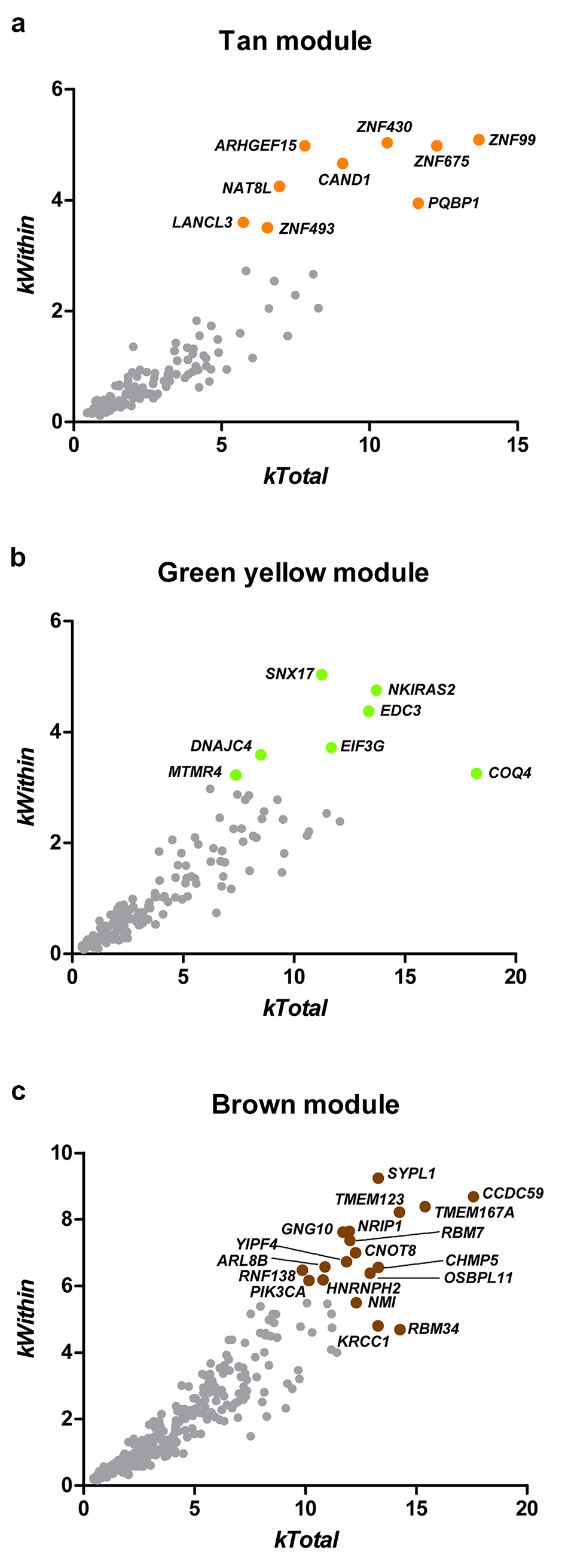

Supplement: S2 Fig — kTotal vs kWithin plots for modules tan (a), green yellow (b), and brown (c). Hubs are identified by colored dots and their respective gene symbols. (TIF) [file pone.0227547.s002.tif]

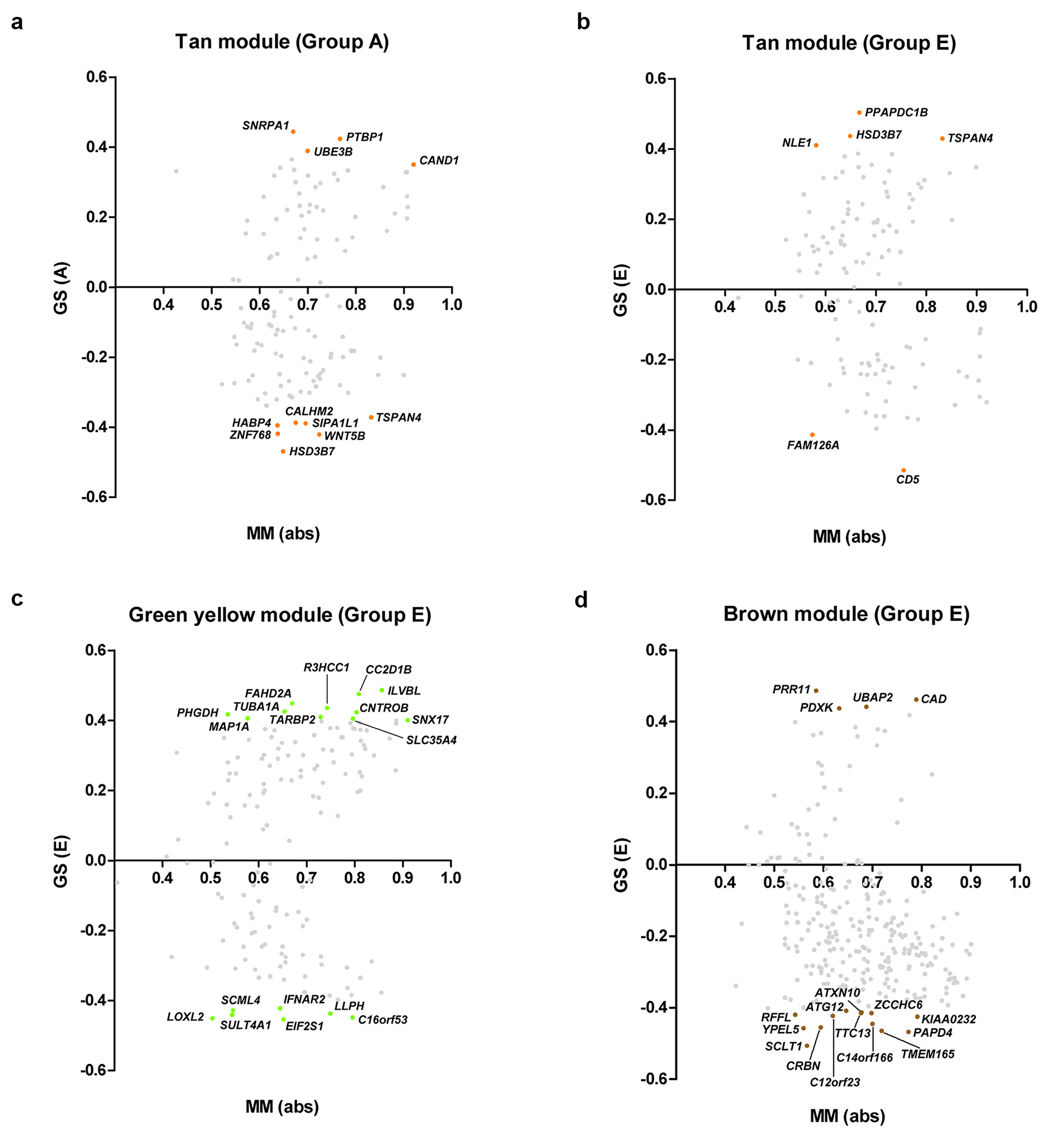

Supplement: S3 Fig — Module Membership (MM) vs. Gene Significance (GS) plots for the module tan (a-b)–groups A and E–and the modules green yellow (c) and brown (d)–group E. Hyperexpressed and hypoexpressed genes for a given trait present positive and negative GS values, respectively. HGS genes are identified by colored dots and their respective gene symbols. (TIF) [file pone.0227547.s003.tif]

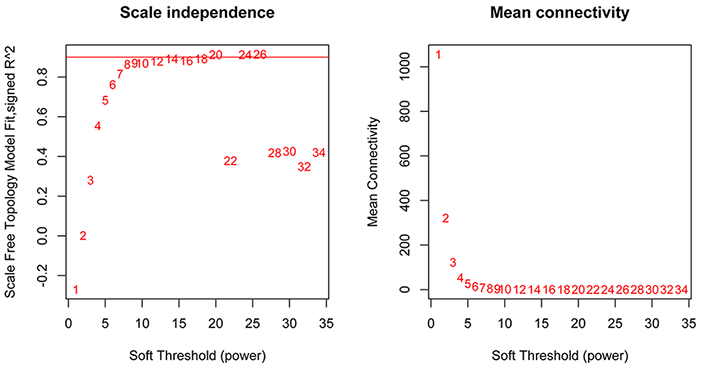

Supplement: S4 Fig — The dataset was fit to a scale-free model of proposed values for β ranging from 1 to 35 (numbers inside the plots). Approximate scale-free topology is attained around soft-thresholding power of 9, which reflects the inflection point where model fit begins to decrease with power increasing (left panel). The plot of the mean connectivity along with the soft-thresholding power (right panel). The red line indicates the scale-free topology R2 fit index cut-off of 0.8700. (TIF) [file pone.0227547.s004.tif]

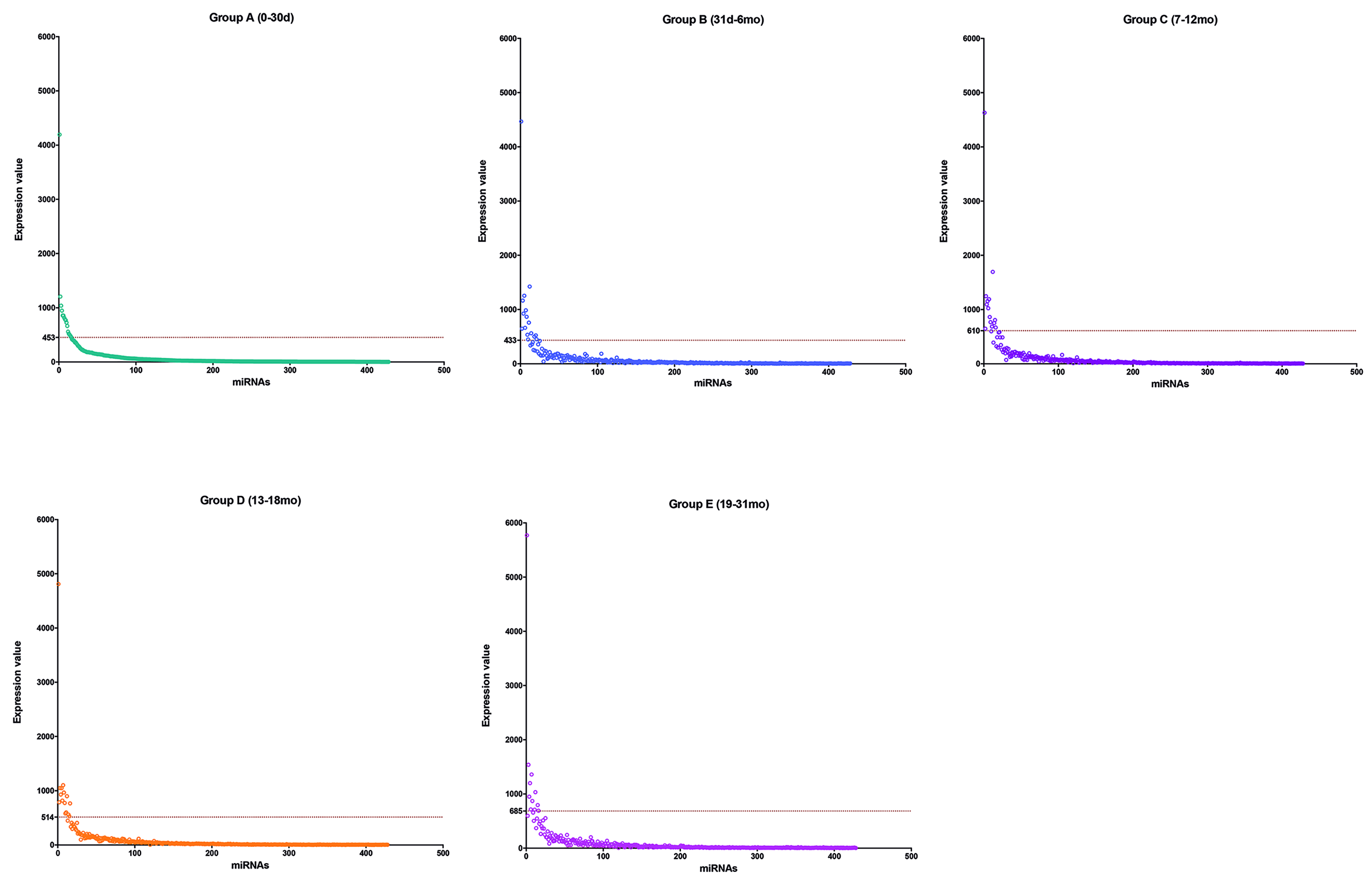

Supplement: S5 Fig — The red dashed lines indicate the cut-off values for selecting abundantly expressed miRNAs for each of the five age groups. (TIF) [file pone.0227547.s005.tif]

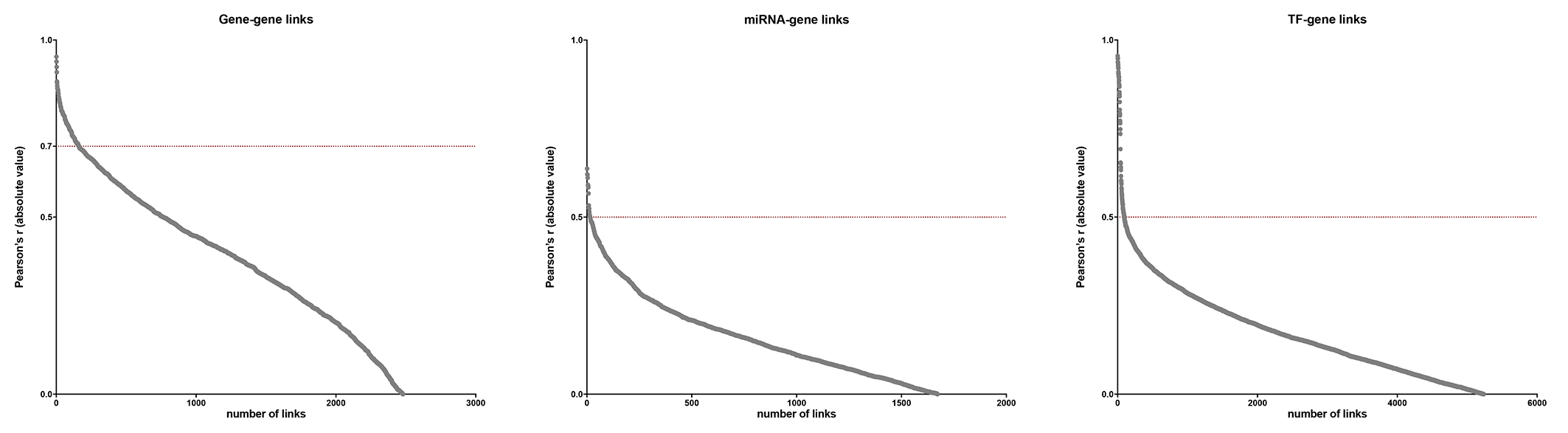

Supplement: S6 Fig — The red dashed lines indicate the cut-off values for selecting link thresholds for gene-gene, miRNA-gene, and TF-gene interactions. (TIF) [file pone.0227547.s006.tif]
